# Hand-foot skin reaction associated with vascular endothelial growth factor receptor tyrosine kinase inhibitors: a FAERS-based pharmacovigilance study

**DOI:** 10.3389/fmed.2026.1796543

**Published:** 2026-06-04

**Authors:** Jinhan Chen, Qian Xu, Bozhou Wang, Huiwen Sun, Qijin Shu

**Affiliations:** 1 The First Affiliated Hospital of Zhejiang Chinese Medical University (Zhejiang Provincial Hospital of Chinese Medicine), Hangzhou, Zhejiang, China; 2 The First School of Clinical Medicine, Zhejiang Chinese Medical University, Hangzhou, Zhejiang, China

**Keywords:** FAERS, hand-foot skin reaction, pharmacovigilance, real-world study, vascular endothelial growth factor receptor tyrosine kinase inhibitors

## Abstract

**Introduction:**

Hand-foot skin reaction (HFSR) is a prevalent and dose-limiting cutaneous toxicity associated with vascular endothelial growth factor receptor tyrosine kinase inhibitors (VEGFR-TKIs), often compromising treatment continuity and efficacy.

**Methods:**

This pharmacovigilance study analyzed reports from the FDA Adverse Event Reporting System (FAERS) database (Q1 2004–Q2 2025). After standard data cleaning, disproportionality analyses using the ROR, PRR, MGPS, and BCPNN were performed to quantify signals for HFSR associated with 11 VEGFR-TKIs. Patient demographics, time-to-onset, and clinical outcomes were also assessed.

**Results:**

A total of 8,668 HFSR reports were analyzed. Eight VEGFR-TKIs generated positive HFSR signals across all four algorithms. Sorafenib and regorafenib showed greater disproportionality. The median time to HFSR onset was 16 days, with most drugs showing an early failure pattern. Subgroup analysis showed that female patients reported stronger signals and had a median onset time (14 days) earlier than male patients (17 days). Hospitalization was the most frequently reported serious outcome.

**Conclusion:**

This large-scale analysis confirmed a significant association between VEGFR-TKIs and HFSR, with significant differences in reported signal values between specific drugs. Proactive, individualized monitoring, particularly during the initial weeks of therapy, is crucial for managing this impactful toxicity.

## 1 Introduction

Vascular endothelial growth factor receptor tyrosine kinase inhibitors (VEGFR-TKIs) represent a well-established class of targeted therapies in oncology and have become a major focus of contemporary anticancer drug research. These inhibitors function by competitively binding to tyrosine kinases, thereby blocking their phosphorylation and selectively inhibiting downstream activation of the VEGFR signaling pathway. This mechanism effectively suppresses tumor-induced neovascularization (1, 2). To date, several agents targeting VEGFRs or related signaling pathways have received regulatory approval worldwide for the treatment of various malignancies (3). Furthermore, numerous clinical investigations have demonstrated that VEGFR-TKIs improve overall prognosis across a range of solid tumors, including lung cancer, hepatocellular carcinoma, colorectal cancer, and renal cell carcinoma (4–7).

Accumulating evidence has reported multi-system adverse events(AEs) associated with VEGFR-TKIs targeted therapies (8). These adverse effects include hypertension, bleeding and thrombosis, hepatic and renal injuries (including proteinuria), hypothyroidism, dermatological alterations, as well as a spectrum of multi-organ toxicities and functional impairments. Of particular clinical significance is the hand-foot skin reaction (HFSR), also referred to as Hand-Foot Syndrome (HFS), palmar-plantar erythrodysesthesia (PPE), or acral erythema, which is recognized as one of the most prevalent and challenging dermatologic toxicities (9). HFSR is characterized by sensory disturbances such as paresthesia (including tingling, burning, dullness, and numbness), localized to the palms and soles, accompanied by edema, erythema, and sometimes nail changes, typically manifesting within the first few weeks of treatment (10). Clinically, HFSR is classified into three grades: Grade 1 involves mild skin changes or dermatitis with paresthesia that does not interfere with daily activities; Grade 2 presents with similar skin alterations accompanied by pain that moderately affects daily functions (e.g., household tasks, phone use), while the skin surface remains largely intact; Grade 3 is marked by ulcerative dermatitis or pronounced skin changes including erythema, swelling, and severe pain, significantly impairing daily activities such as walking, eating, dressing, and moving, and may involve complications such as desquamation, blistering, edema, bleeding, and hyperkeratosis. Empirical studies have demonstrated a correlation between the cumulative dose of sorafenib and the incidence of Grade 2–3 HFSR (11). The development of HFSR adversely affects drug tolerability, often necessitating dose reductions or treatment interruptions, thereby compromising patients’ ability to perform routine activities and ultimately diminishing their quality of life (12).

The FDA Adverse Event Reporting System (FAERS) is recognized as the most important spontaneous reporting database worldwide and plays a crucial role in post-marketing drug safety surveillance. By applying advanced data mining methods, FAERS facilitates the quantitative assessment of potential associations between drugs and AEs, thereby supporting pharmacoepidemiological investigations and pharmacovigilance efforts (13, 14).

## 2 Methods

### 2.1 Data source and collection

ASCII-formatted datasets obtained from the FAERS database were imported into R statistical software (version 4.4.2) for data integration, cleaning, and standardization. Duplicate entries in the DEMO table were removed according to the deduplication guidelines prescribed by the FDA. Following the FDA-recommended method for removing duplicate reports, the PRIMARYID, CASEID, and FDA_DT fields were selected from the DEMO table. The data were sorted by CASEID, FDA_DT, and PRIMARYID. For reports with the same CASEID, the report with the highest FDA_DT value was retained, as a higher value indicates a newer report date. For reports with identical CASEID and FDA_DT values, the report with the largest PRIMARYID value was retained. Subsequently, all data were further deduplicated by cross-referencing the PRIMARYID field across six additional tables, including the DRUG table. Reports containing misspelled drug names, duplicate records, or AEs unrelated to the study were excluded. AEs were then standardized using preferred terms (PT) from the Medical Dictionary for Regulatory Activities (MedDRA), version 27.1, and categorized according to System Organ Class (SOC).

### 2.2 Data extraction and statistics

**Figure 1:**
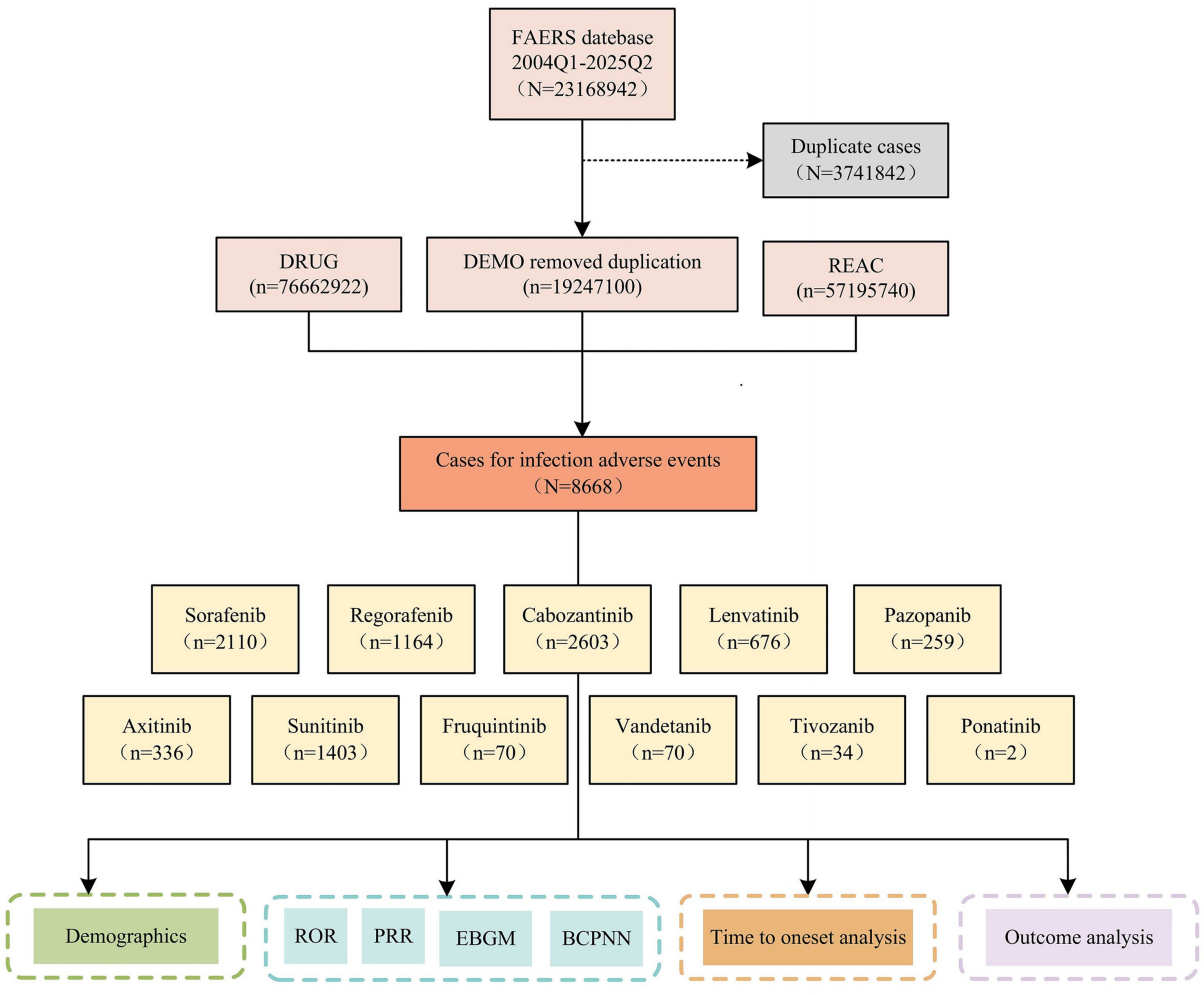
The flow chart of the study.

Eleven VEGFR-TKIs—sorafenib, regorafenib, cabozantinib, sunitinib, axitinib, lenvatinib, vandetanib, pazopanib, fruquintinib, tivozanib, and ponatinib were included in the analysis. To ensure comprehensive data collection, we searched for all drug names (including generic names, brand names, and active ingredient names) reported to the FAERS database using the National Center for Biotechnology Information (NCBI) and FDA-registered Drugs (Drugs@FDA). In addition, we collated demographic information of patients who experienced adverse reactions to these 11 VEGFR-TKIs retrieved from the FAERS database. After identifying cases using all relevant drug names (active ingredients, brand names, and salt forms), only reports in which a VEGFR-TKI was designated as the “primary suspect (PS)” drug were included. The data extraction process is shown in Figure 1. There were no privacy concerns and no ethical review required for this study.

### 2.3 Analysis methods

Four distinct methods were used to assess the signal strength of AEs: the reporting odds ratio (ROR), proportional reporting ratio (PRR), Bayesian confidence propagation neural network (BCPNN), and multi-item gamma Poisson shrinker (MGPS) (14, 15). The corresponding formulas and evaluation criteria are detailed in Supplementary Table 1. Higher values indicate a stronger reporting signal between a specific drug and HFSR. Furthermore, the onset time of drug-induced HFSR was assessed by calculating the interval between the initiation date of the primary suspected drug and the date of the first reported AE(s). Based on the Weibull shape parameter (*β*) and its 95% confidence interval (CI), adverse event patterns are defined as follows: Early failure pattern: *β* < 1 and upper 95% CI < 1, characterized by an initial rise in risk followed by a decline. Wear-out failure pattern: *β* > 1 and lower 95% CI > 1, indicating a progressive increase in risk over time. Random failure pattern: *β* = 1 or 95% CI includes 1, reflecting a constant risk throughout treatment. Additionally, the cancer types within the drug-exposed population were categorized and characterized.

## 3 Results

### 3.1 Clinical characteristics

**Figure 2:**
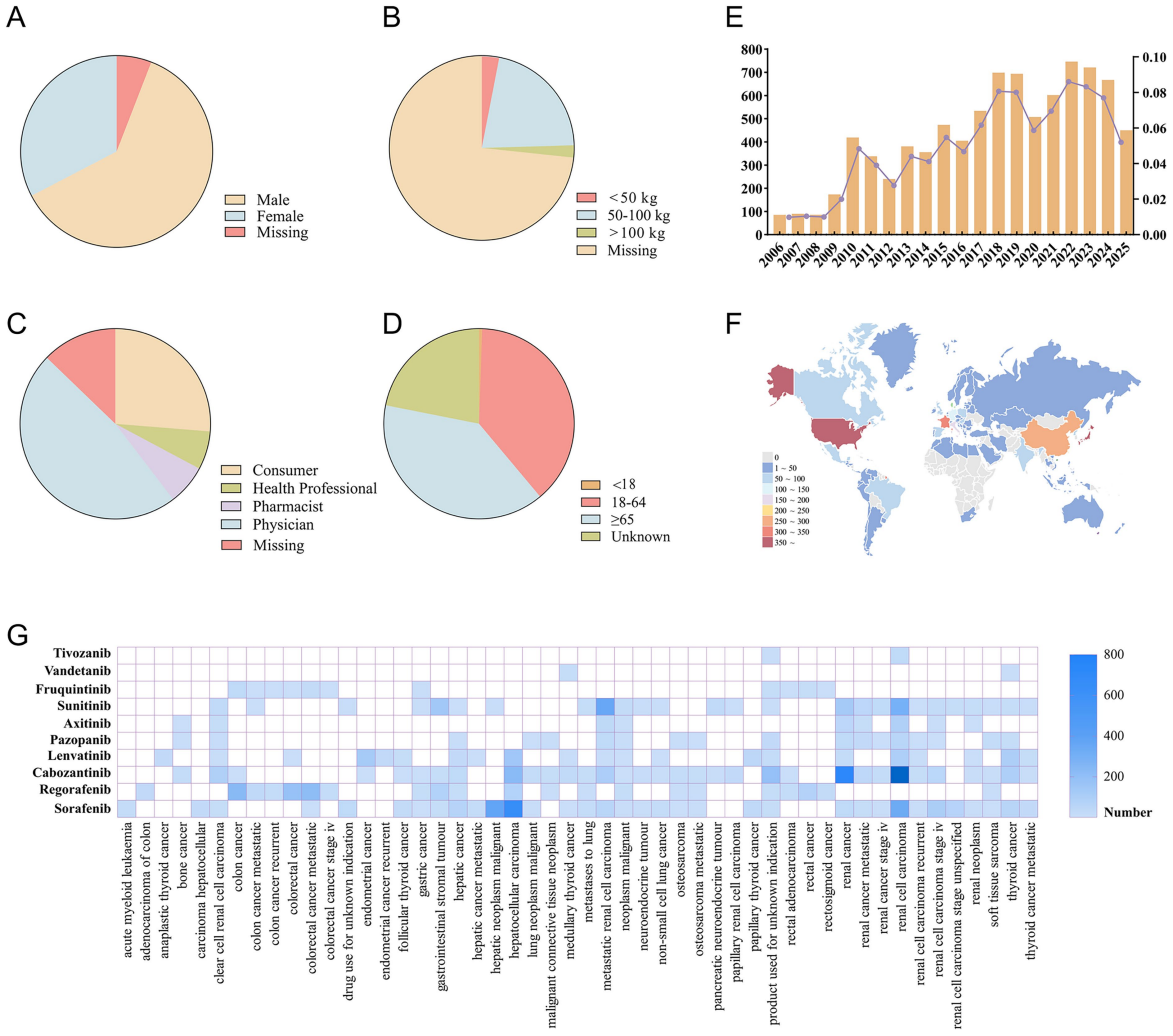
AE reports related to HFSR induced by VEGFR-TKIs in the FAERS database. **(A)** Gender distribution of patients. **(B)** Weight distribution of the patients. **(C)** Distribution of reporters by occupation. **(D)** Age distribution of patients. **(E)** Annual trends in AE reports. **(F)** Report countries distribution of the patients. **(G)** Indications distribution of the patients.

**Figure 3:**
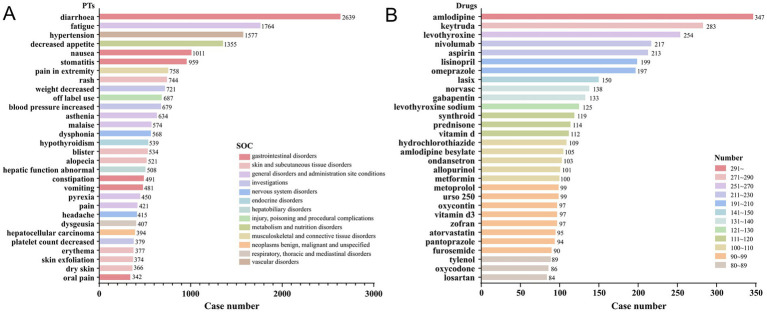
**(A)** Distribution of combined medications, different colors represent different SOC. **(B)** Distribution of combined medications.

A total of 8,668 AE cases associated with VEGFR-TKIs were identified from the FAERS database. The clinical characteristics of the included patients are summarized in Figure 2 (Supplementary Table 2). Demographic analysis indicated that females accounted for 32.6% (*n* = 2,830) of total cases, while males accounted for 61.5% (*n* = 5,328). Except for lenvatinib, males represented a higher proportion for all other drugs. Patient ages predominantly fell into two groups: 18–64 years (38.4%) and ≥65 years (39.2%). Geographically, the top five countries by number of reported cases were the United States (37.7%), Japan (32.4%), China (5.1%), France (3.5%), and Italy (1.8%). Physician-submitted reports accounted for the largest proportion (47.6%). Primary indications for patients were liver cancer, kidney cancer, gastrointestinal tumors, and thyroid cancer, aligning with the cancer types treated by these VEGFR-TKIs. Analysis of concomitant medications showed that amlodipine as the most frequently used drug, followed by other antihypertensives such as ramipril, furosemide, and metoprolol. Immune checkpoint inhibitors, including pembrolizumab and nivolumab, ranked second. Analysis of concomitant adverse reactions identified several noteworthy events, including rash, stomatitis, elevated blood pressure, and abnormal liver function. Concomitant adverse reactions primarily affected the skin, cardiovascular, gastrointestinal and hepatobiliary systems (Figure 3).

### 3.2 AE signal calculation and subgroup results

**Figure 4:**
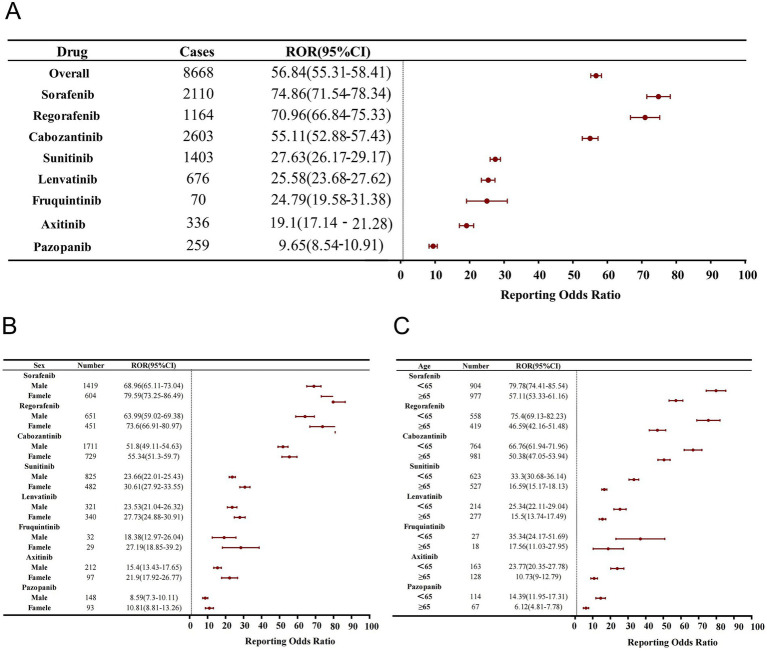
**(A)** ROR forest plots overall versus individual drugs. **(B)** ROR forest plots for individual drug by gender subgroup. **(C)** ROR forest plots for individual drug by age subgroup.

To further assess the reporting disproportionality of HFSR associated with the included VEGFR-TKIs, we conducted a comprehensive AE signal detection analysis, with results presented in Supplementary Table 3. Except for extremely small subgroups including ponatinib (*n* = 2), vandetanib (*n* = 11) and tivozanib (*n* = 34), all four detection algorithms identified positive signals for HFSR associated with the remaining eight VEGFR-TKIs. Sorafenib and regorafenib exceeded the overall ROR threshold. Although positive signals were found in vandetanib and tivozanib, they were only used as a reference for exploration in this study due to small and unstable samples. Furthermore, a review of the prescribing information for these drugs revealed that HFSR is listed as a significant skin toxicity. As current antitumor regimens increasingly shift toward precision and individualized management, we explored age and gender subgroups to investigate differences in HFSR-related reporting disproportionality across different drugs. Results showed that in the gender subgroup, female patients exhibited higher ROR than male patients for all VEGFR-TKIs except vandetanib. In the age subgroup, similarly, except for vandetanib, the ROR was higher in patients aged <65 years than in those aged ≥65 years (Figure 4).

### 3.3 Time-to-onset and outcome results

**Figure 5:**
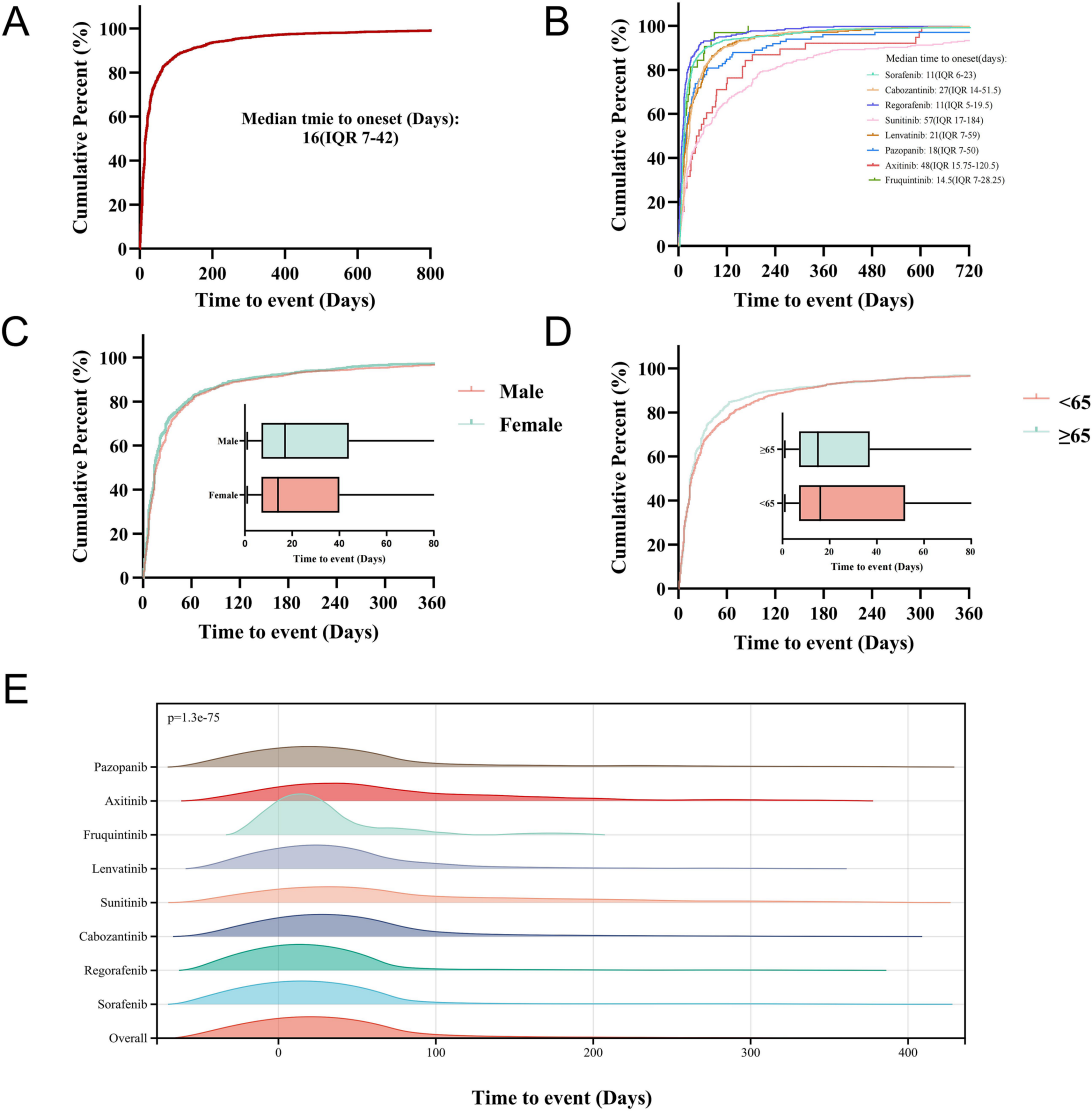
**(A)** Overview of median onset times for HFSR associated with VEGFR-TKIs. **(B)** Cumulative curve of time to onset for different VEGFR-TKIs. **(C)** Cumulative plots of time to onset according to gender subgroups. **(D)** Cumulative plots of time to onset according to age subgroups. **(E)** Mountain plot of onset time of different VEGFR-TKIs.

Time-to-onset analysis was performed for 2,928 HFSR patients with complete onset time data (33.78% of the cohort) (Figure 5) (Supplementary Table 4). The median time to HFSR onset was 16 days (interquartile range, IQR: 7–42 days). Statistically significant differences in onset time were observed among different VEGFR-TKIs. Sorafenib and regorafenib demonstrated the earliest median onset time of 11 days. Sunitinib and ponatinib exhibited the latest median onset time of 57 days. Notably, ponatinib, vandetanib, and tivozanib were excluded from this analysis due to unreliable results in very small subgroups. When stratified by gender and age, the median onset time was 14 days (IQR: 7–40 days) in females, which was earlier than the 17 days (IQR: 7–44 days) observed in males. The median time to onset in the ≥65 age group was 15 days (IQR: 7–40 days), which was earlier than the 16 days (IQR: 7–52 days) observed in the <65 age group. In the subgroup populations, the time to onset was similar to the overall median time to onset, with no significant differences. The Weibull distribution model further revealed that most HFSR cases conformed to an early failure pattern (shape parameter *β* < 1 and upper 95% CI < 1), indicative of a time-dependent reduction in HFSR risk (Supplementary Table 2).

**Figure 6:**
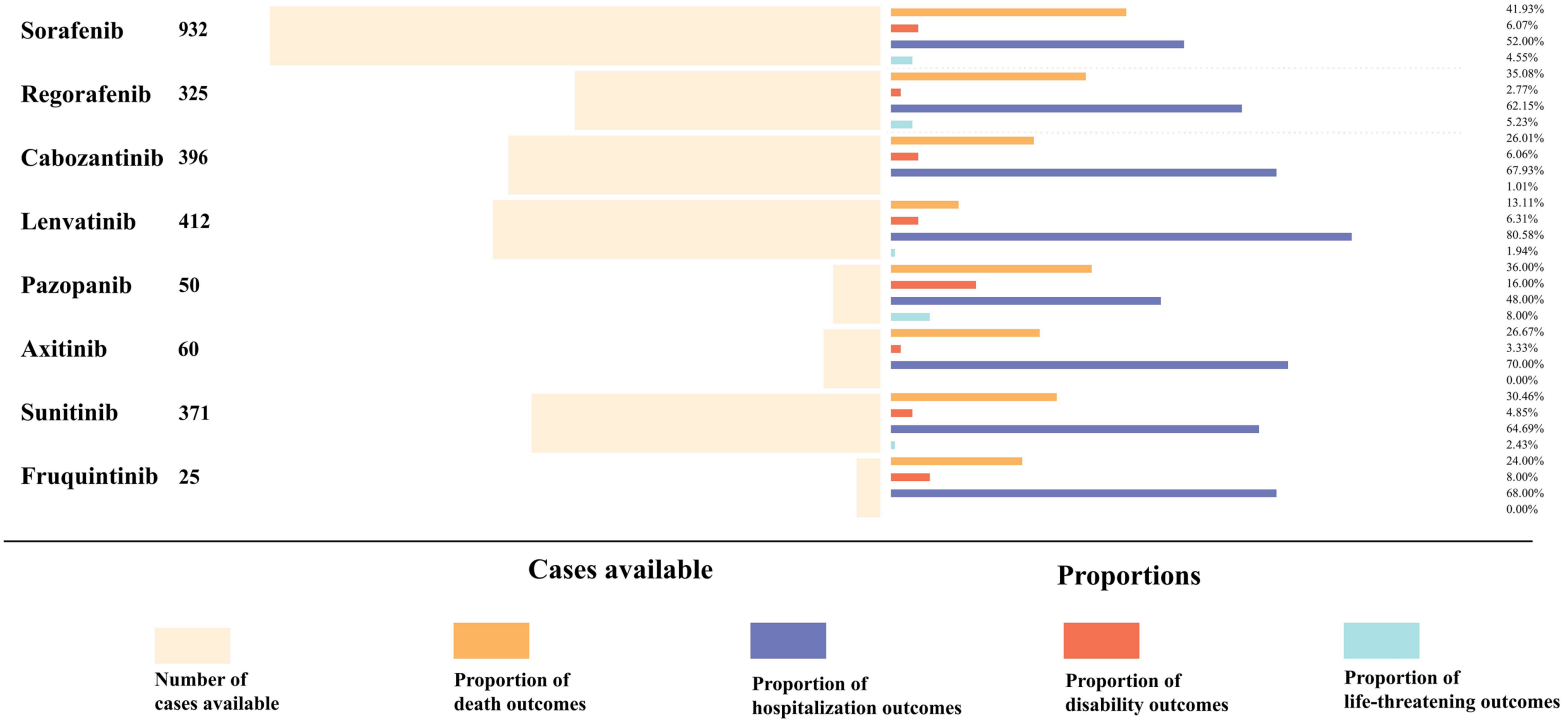
Outcome analysis of VEGFR-TKIs-related HFSR. The number of cases, hospitalizations, and fatality proportions for VEGFR-TKIs-associated HFSR were visualized.

To determine the severity of clinical outcomes associated with VEGFR-TKIs in HFSR, we analyzed the proportion of outcomes (including death, hospitalization, life-threatening events, and disability) across the different VEGFR-TKI agents (Figure 6). Hospitalization was the most common outcome in VEGFR-TKI-related HFSR. After excluding ponatinib, vandetanib, and tivozanib in the small sample group, lenvatinib had the highest hospitalization rate at 80.58%, followed by axitinib (70%). Differences in mortality outcomes were also observed, with higher rate, sorafenib (41.93%), pazopanib (36%), and regorafenib (35.08%), whereas lenvatinib (13.11%) had the lowest mortality rates.

## 4 Discussion

With the continuous rise in new cancer cases worldwide, public awareness of adverse reactions induced by anticancer drugs has increased. HFSR triggered by anticancer drugs has become a significant barrier to sustainable cancer treatment, often leading to dose reduction or treatment discontinuation. Previous studies on HFSR associated with VEGFR-TKIs have primarily focused on clinical trials and meta-analyses, lacking comprehensive investigations in the broader post-marketing population. To address the limitations of clinical trials and case reports about HFSR associated with VEGFR-TKIs, this study utilized the FAERS database, and employed four disproportionate risk analysis methods—ROR, PRR, BCPNN, and MGPS—to quantify the association between VEGFR-TKIs and HFSR. Higher signal detection values indicate stronger correlations and stronger reporting disproportionality for HFSR among cancer patients receiving these drugs. We systematically analyzed 8,668 HFSR-related reports involving 11 VEGFR-TKIs as primary suspected drugs. We described baseline patient demographics and discussed temporal differences in HFSR onset associated with these inhibitors. Additionally, we systematically summarized outcomes in the HFSR cohort and explored risks across gender and age subgroups.

This study found that the frequency distribution of HFSR associated with VEGFR-TKIs was similar between individuals under 65 years of age (38.4%) and those aged 65 years and above (39.2%), indicating the broad age distribution of HFSR. From a gender perspective, male patients outnumbered female patients, potentially reflecting the significantly larger male cohort in the FAERS database. Regarding reporter distribution, physicians accounted for 47.6%, while health professionals and pharmacists represented 6.5 and 6.8%, respectively. Overall, healthcare professionals contributed over half of the reports, providing some support for data reliability. Geographically, most reports originated from the United States, Japan, China, and Germany. Notably, despite the majority of drug reports originating from the United States, sorafenib exhibited a high distribution in the Asia-Pacific region. Studies indicate that Asian patients with hepatocellular carcinoma treated with sorafenib exhibit a significantly higher incidence of HFSR than those in other regions (16). This phenomenon may stem from racial differences in genetic polymorphisms of the TNF-*α*, VEGF, and UGT1A9 genes (17). Finally, the distribution of indications aligns with approved therapeutic uses, primarily focusing on liver cancer, kidney cancer, gastrointestinal tumors, and thyroid cancer.

The high frequency of cardiovascular drugs and cardiovascular events among patients receiving concomitant medications and experiencing adverse reactions highlights the significant impact of VEGFR-TKIs on the cardiovascular system. Clinicians are advised to monitor blood pressure changes during adverse reaction management and to carefully review patients’ histories of chronic hypertension treatment. Another important class of concomitant medication is immune checkpoint inhibitors, which also induce HFSR. Studies have shown that immune checkpoint inhibitors have significant skin toxicity. The underlying mechanism may involve T-cell activation against shared antigens present in both tumor cells and normal tissues, along with increased release of immune-related effector cytokines and antibodies (18). Therefore, the combination of targeted therapy and immunotherapy may be one of the important reasons for the high incidence of HFSR. Furthermore, it is noteworthy that patients experiencing HFSR are at heightened risk for other cutaneous and mucosal toxicities, including rash, alopecia, and oral mucositis. These effects are associated with shared toxicities at the target sites, resulting from concurrent inhibition of the VEGF signaling pathway in the skin, mucosa, and associated appendages. This inhibition leads to microvascular dysfunction and impaired epithelial repair (19).

In pharmacovigilance analyses, eight VEGFR-TKIs induced HFSR with positive signals across all four disproportionality algorithms. Sorafenib and regorafenib, two VEGFR-TKIs characterized by low selectivity and high potency, demonstrated the strongest association with HFSR, exceeding the overall ROR threshold. This finding was further confirmed in a meta-analysis assessing high-level HFSR risk: sorafenib (RR = 30.72; 95% CI: 19.27–48.97) and regorafenib (RR = 28.91; 95% CI: 10.08–82.90) exhibited significantly higher relative risk than other agents (20). Cabozantinib, lenvatinib, and sunitinib—also multi-targeted VEGFR-TKIs with lower selectivity—ranked next in association strength. Conversely, moderately to highly selective VEGFR-TKIs such as fruquintinib, axitinib, pazopanib showed relatively weaker associations with HFSR.

“Off-target effects” refer to the unintended binding or inhibition of molecules other than the intended therapeutic target by a drug, leading to unexpected biological consequences, including toxicity. Based on previous studies, it can be speculated that the cascade of signal intensity observed in this study may reflect “off-target effects” of less selective VEGFR-TKIs. Some VEGFR-TKIs, acting as multi-kinase inhibitors, simultaneously affect a range of known vascular endothelial protective factors beyond VEGFR inhibition. These include the fibroblast growth factor receptor (FGFR) family, the platelet-derived growth factor receptor (PDGFR) family, rearranged during transfection (RET), and rapidly accelerated fibrosarcoma (RAF) kinases. For example, studies have shown that sorafenib has inhibitory effects on PDGFR, VEGFR, c-KIT, and RAF, which may induce the development of HFSR through one or more of these receptors or pathways (21). Similarly, regorafenib blocks multiple receptors involved in the regulation of tumor angiogenesis (VEGF-1,-2,-3), carcinogenesis (RET, RAF-1), and the tumor microenvironment (PDGFR and FGFR) (22). Studies have found that inhibition of VEGFR and PDGFR may prevent vascular repair mechanisms from functioning normally, resulting in repeated subclinical trauma in high-pressure areas such as the palms and soles (23). Thus, combined inhibition of these receptors appears to be critical, since inhibition of PDGFR (imatinib) or VEGF(bevacizumab) alone is not strongly associated with the development of HFSR (24). Taken together, the presence of “off-target effects” may explain the more pronounced HFSR observed with less selective VEGFR-TKIs. Although these potential mechanisms have been emphasized, multiple other factors may also influence outcomes. Research indicates that the accumulation of VEGFR-TKIs in keratinocytes is a key mechanism underlying the occurrence of HFSR. The transport mechanisms of drugs in the skin differ from those in other organs, and these differences influence drug uptake and excretion, leading to variations in drug concentration across the skin (25). For example, sorafenib increases its accumulation in keratinocytes via organic anion transporter 6, thereby triggering HFSR (26). It should be emphasized that all of the above mechanistic discussions, including off-target effects and keratinocyte drug accumulation, were derived solely from published external studies and were not investigated further in the present work. These hypotheses were not verified by our data and require further experimental confirmation. More robust evidence is needed to clarify the potential differences in the mechanisms of VEGFR-TKI-induced HFSR.

In our analysis of median time to onset, we found eight VEGFR-TKIs except Fruquintinib exhibited an early failure pattern, consistent with findings from clinical research (27). The overall median time to onset showed little variation by gender or age, with consistent results across subgroups and the overall population, generally occurred around 2 weeks. Hand-foot skin reactions are generally considered an early-onset adverse reaction to targeted therapy. The summary and visualization of median onset times suggest that early attention should be paid to this adverse reaction and implement management and that intervention promptly if it occurs. However, a limitation of this analysis is that time-to-onset data were only available for a subset of HFSR cases (2,928/8,667; 33.78% of the cohort), which may not be fully representative of all reported HFSR events.

Disproportionality analysis revealed higher ROR values for HFSR in individuals aged <65 years across all eight VEGFR-TKIs. A meta-analysis confirmed the risk of HFSR in VEGFR-TKI-treated patients across different age groups, demonstrating a high-risk RR for patients under 60 years of age in both all-grade and high-grade HFSR (RR = 8.98; 95% CI: 5.72–14.10) and (RR = 23.75; 95% CI: 15.84–35.62) compared with those aged ≥ 60 years (RR = 5.81; 95% CI: 4.12–8.19 and RR = 16.12; 95% CI: 7.31–35.53) (20). Notably, although the <65 age group showed higher disproportionality (ROR), the ≥65 age group exhibited a slightly earlier median onset time (15 days vs. 16 days). These two observations are not directly comparable: ROR reflects the strength of reporting disproportionality, whereas time-to-onset reflects temporal pattern of adverse event reports. Earlier onset does not imply higher clinical risk, nor does a higher ROR necessarily translate to higher incidence or earlier onset. The present study did not collect individual-level incidence data; therefore, we cannot determine whether the observed differences in onset time and disproportionality represent true age-related biological differences or reflect reporting biases. Gender subgroup analysis revealed that female exhibited higher ROR values than male across all nine VEGFR-TKIs. Clinical studies of sorafenib identified female gender as a significant independent risk factor for Grade ≥ II HFSR (27). This may relate to pharmacokinetic differences and variations in skin structure and physiology, though the specific gender-related mechanisms underlying HFSR remain unclear. Furthermore, clinical studies have identified elevated serum folate and red blood cell folate levels, high body mass index, elevated baseline ALT/AST levels, excessive sweating, and a history of gallstones as risk factors for moderate-to-severe HFSR (28). Additionally, HFSR is a dose-limiting toxicity, with its incidence and severity closely related to the administered dose, cumulative dose, dosing frequency, and regimen. Furthermore, the incidence of HFSR in patients receiving sorafenib plus bevacizumab was more than double that of sorafenib monotherapy (79% vs. 31%, *p* < 0.05) (11). Therefore, risk-stratified management of patients with targeted therapy-induced HFSR is necessary, with heightened vigilance for HFSR in high-risk populations.

Our findings supports a risk stratification approach for prevention and management of HFSR in patients receiving VEGFR-TKIs. Significant variability across different drugs, regimens, and patient populations necessitates individualized, precision-based monitoring strategies. Heightened vigilance is required for high-risk agents such as sorafenib and regorafenib, especially when combined with immunotherapy. The observation that the vast majority of HFSR cases occur within 2–3 weeks identifies this period as a critical window for patient education and clinical assessment. This supports proactive management strategies to mitigate HFSR’s impact on antitumor efficacy and preserve the benefits of targeted therapy.

For mild cases of HFSR, it is recommended to minimize exposure to risk factors such as mechanical friction, tight-fitting shoes or socks, and elevated temperatures. Treatment typically involves cold compresses and topical medications (including urea ointment) to alleviate symptoms (29). For moderate to severe HFSR (grade 2 or higher), celecoxib or topical diclofenac-both cyclooxygenase-2 (COX-2) inhibitors-may be used for prevention and treatment (30). For patients experiencing intolerable or severe HFSR, reducing the dose or temporarily discontinuing targeted therapy is advised, along with consideration of alternative medications associated with a lower incidence of HFSR (31). Studies also indicate zinc supplementation may elicit a therapeutic response for HFSR (32, 33). Recently, complementary and alternative therapies have emerged as potential adjunctive approaches for HFSR induced by targeted therapies, offering new perspectives for clinical management (34). Therefore, combining early risk identification with effective drug management or supportive measures following HFSR onset will lead to improved symptom control in patients and enhanced antitumor efficacy.

However, this study has several limitations that should be considered when interpreting its findings. First, as a pharmacovigilance study based on the FAERS database, this analysis is signal detection research rather than a cohort study or formal risk assessment. FAERS is a spontaneous reporting system, which entails inherent biases, including underreporting, selective reporting (e.g., over-reporting of serious or novel AEs), and duplicate reports. These factors preclude the calculation of AE incidence rates because the total number of patients exposed to each drug (denominator) is unavailable. Consequently, disproportionality measures such as the ROR reflect the strength of reporting associations, not clinical risk or causality. Second, the FAERS database lacks essential clinical information, including baseline patient characteristics (e.g., comorbidities, concomitant medications, performance status), drug exposure duration, and dosage details. As a result, we could not adjust for potential confounders (e.g., disease severity, prior treatments, and drug–drug interactions), which may influence both HFSR occurrence and reporting patterns. Third, while we applied a standardized data cleaning approach to remove duplicate entries, complex duplicates and unverifiable reports may still remain. Additionally, the quality and completeness of reports vary across different countries and healthcare systems, introducing further heterogeneity. Fourth, our time-to-onset analysis was restricted to the subset of reports with available onset dates (33.78% of the cohort), which may not be representative of all HFSR cases. The Weibull distribution model provides descriptive insights into temporal patterns but does not establish causal mechanisms. Despite these limitations, the FAERS database remains a valuable data source for generating safety signals in large, real-world patient populations. Our study generates hypotheses regarding differential reporting disproportionality of HFSR across VEGFR-TKIs and patient subgroups. These findings should be interpreted as hypothesis-generating and require confirmation through well-designed prospective cohort studies or pooled analyses of clinical trials that can properly adjust for confounding and calculate true incidence rates.

## 5 Conclusion

This study quantifies the strong association between VEGFR-TKIs and HFSR using FAERS data. Sorafenib and regorafenib showed the strongest reporting disproportionality among the agents assessed. Female patients were associated with higher reporting disproportionality, suggesting they may be potential factors influencing HFSR reporting patterns. The median time to onset was approximately 16 days, with most cases following an early failure pattern. The findings emphasize the need for early monitoring and individualized prevention strategies in clinical practice to manage this dose-limiting toxicity.

## Data Availability

The original contributions presented in the study are included in the article/Supplementary material, further inquiries can be directed to the corresponding author.
