# Supplementary material for: Hand-foot skin reaction associated with vascular endothelial growth factor receptor tyrosine kinase inhibitors: a FAERS-based pharmacovigilance study
Source: Front Med (Lausanne). 2026 Jun 4;13:1796543. doi: 10.3389/fmed.2026.1796543 (PMC13275275; doi:10.3389/fmed.2026.1796543)
Supplement: Supplementary file 1 [file Table_1.DOCX]

**Supplementary Table 1. The corresponding formulas and evaluation criteria of ROR, PRR, BCPNN, MGPS.**

| Algorithms | Formulas | Criteria |
| --- | --- | --- |
| ROR | 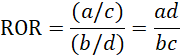  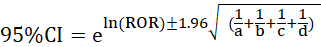 | a ≥ 3, The lower limit of 95% CI (RORL) ＞ 1 |
| PRR | 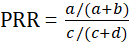  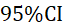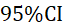=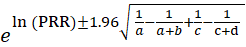 | a ≥ 3, PRR ≥ 2, χ² ≥ 4 |
|  | 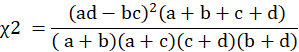 |  |
| BCPNN | *α_i_*=*β_j_*=1,*α*=*β*=2,*γ_ij_*=1  *N*＝*a*+*b*+*c*+*d* | a ≥ 3, The lower limit of 95% CI (IC025) ＞ 0 |
|  | $\text{IC}=\log_{2}\frac{a\text{(}a+b+c+d\text{)}}{\text{(}a+b\text{)(}a+c\text{)}}$ $\text{E(IC)}=\log_{2}^{^{\frac{\text{(}a\text{+}\gamma_{ij}\text{)(}N\text{+}\alpha\text{)(}N\text{+}\beta\text{)}}{\text{(}N\text{+}\gamma\text{)(}a\text{+}b\text{+}\alpha_{i}\text{)(}a\text{+}c\text{+}\beta_{j}\text{)}}}}$    V(IC)= $\text{SD}=\sqrt{\text{V(IC)}}$   IC025=E(IC)-2SD |  |
|  |  |  |
| MGPS | 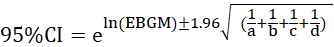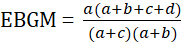  EBGM05= | EBGM05 > 2 |
