# Supplementary material for: Hand-foot skin reaction associated with vascular endothelial growth factor receptor tyrosine kinase inhibitors: a FAERS-based pharmacovigilance study
Source: Front Med (Lausanne). 2026 Jun 4;13:1796543. doi: 10.3389/fmed.2026.1796543 (PMC13275275; doi:10.3389/fmed.2026.1796543)
Supplement: Supplementary file 3 [file Table_3.DOCX]

**Supplementary Table 3. Detection results of AE signals (four algorithms).**

| Drugs | Case reports | ROR(95%CI) | PRR(95%CI) | X2 | EBGM(EBGM05) | IC(IC025) |
| --- | --- | --- | --- | --- | --- | --- |
| Overall | 8668 | 56.84(55.31-58.41 ) | 56.11(54.61-57.64) | 282912.42 | 34.22(33.3) | 5.1(5.05) |
| Sorafenib | 2110 | 74.86(71.54-78.34 ) | 73(69.83-76.31) | 135406.57 | 66.04(63.11) | 6.05(5.93) |
| Regorafenib | 1164 | 70.96(66.84-75.33) | 69.21(65.28-73.36) | 74098.93 | 65.57(61.76) | 6.03(5.87) |
| Cabozantinib | 2603 | 55.11(52.88,57.43) | 54.13(51.97,56.37) | 119586.3 | 47.79(45.86) | 5.58(5.49) |
| Sunitinib | 1403 | 27.63(26.17-29.17) | 27.37(25.93-28.88) | 33364.71 | 25.67(24.32) | 4.68(4.58) |
| Lenvatinib | 676 | 25.58(23.68-27.62) | 25.35(23.49-27.36) | 15326.21 | 24.59(22.77) | 4.62(4.46) |
| Fruquintinib | 70 | 24.79(19.58-31.38) | 24.57(19.45-31.03) | 1578.12 | 24.49 (19.35) | 4.61(3.86) |
| Tivozanib | 34 | 22.07(15.74-30.93) | 21.89(15.66-30.6) | 677.03 | 21.86 (15.59) | 4.45(3.29) |
| Axitinib | 336 | 19.1(17.14-21.28) | 9.62(8.51-10.81) | 5632.78 | 18.69(16.77) | 4.22(3.99) |
| Pazopanib | 259 | 9.65(8.54-10.91) | 9.62(8.51-10.87) | 1977.69 | 9.52(8.42) | 3.25(3.02) |
| Vandetanib | 11 | 5.33(2.95-9.63) | 5.32(2.95,9.6) | 38.57 | 5.32 (2.94) | 2.41(1.13) |
| Ponatinib | 2 | 0.13(0.03-0.52 ) | 0.13(0.03-0.52) | 11.61 | 0.13 (0.03) | -2.94(-4.11) |
